# Three-Dimensionally Conformal Porous Polymeric Microstructures of Fabrics for Electrothermal Textiles with Enhanced Thermal Management

**DOI:** 10.3390/polym10070748

**Published:** 2018-07-06

**Authors:** Su Liu, Jianliang Gong, Bingang Xu

**Affiliations:** 1 Engineering Research Center of Technical Textile, Ministry of Education, Shanghai 201620, China; 2 Nanotechnology Center, Institute of Textiles and Clothing, The Hong Kong Polytechnic University, Hung Hom, Kowloon, Hong Kong, China; jl.gong@polyu.edu.hk; 3 College of Textiles, Donghua University, 2999 North Renmin Road, Songjiang District, Shanghai 201620, China

**Keywords:** modified fabric, polymer, 3D conformal porous microstructure, electrothermal textile, enhanced thermal management

## Abstract

Three-dimensionally conformal porous microstructured fabrics (3CPMFs) are a new kind of modified fabrics with three-dimensionally conformal porous microstructures of introduced materials recently developed for wearable technology. They can effectively introduce customized functional performance based on the choice of brick materials, while at the same time maintain the excellent inherent properties of textiles. In this paper, based on the introduction of polystyrene with low thermal conductivity at only 8 × 10^−4^ g cm^−2^, we developed a kind of polyester fabric-based 3CPMF with enhanced thermal insulation, while maintaining its unique fabric texture, flexibility, moisture permeability, and light weight. It was demonstrated to be a good textile material for the fabrication of wearable electrothermal textile (ET) devices with enhanced thermal management. Compared to pristine fabric-based ET devices, this kind of 3CPMF-based ET devices can obtain higher temperatures under the same input power to provide thermal comfort for human beings, while saving more electric power to achieve the same thermal equilibrium temperature. We believe that, based on the choice of different functional materials and textiles, a wide range of 3CPMFs with customized functionalities and properties can be designed and developed for the realization of a brand-new class of truly wearable devices with desired functional performance and daily garment-like safety and comfort.

## 1. Introduction

Electrothermal textiles (ETs) are a kind of smart electronic device mainly based on Joule heating for the thermal management of target objects [1]. One of their significant applications is to provide thermal comfort. They can also be designed to function as desired garments or accessories to actively warm up target objects, regardless of the degree of chilly weather, by controlling the input power. To be truly put on human bodies like normal textiles, ETs must, first and foremost, be safe and possess the comfort properties of textiles. A common strategy is to embed flexible conductive materials into textile materials by knitting or weaving the ETs with a loose structure [2,3]. This kind of treatment can make the wearers feel comfortable like they are wearing common clothes, but at the same time it inevitably results in a low heating efficiency for the ETs due to high emissivity. To improve the efficiency of ETs, an alternative strategy is to introduce conductive nanowires (e.g., carbon nanotubes or silver nanowires) onto the surface of fabrics by dip coating them, in order to decrease the pore size of the fabric textures [4,5,6]. This approach can endow common fabrics with Joule heating capability, and effectively decrease the human body radiation by reflection while maintaining the water vapor permeability. The introduced nanomaterials, by simple surface deposition, however, may give rise to underlying safety and health problems for wearers.

In our previous work, we have innovatively developed a nature-inspired approach of using breath figures (BFs) for direct surface modification of textile substrates with novel three-dimensionally conformal porous microstructures and showed their promising applications in the incorporation of functional materials for the photocatalytic degradation of pollutants [7]. We have also successfully explored the BF approach for a molding synthesis of hierarchical polymer micro lens arrays for energy harvesting [8]. In this paper, we design and develop a unique kind of desired textile, i.e., three-dimensionally conformal porous microstructured fabrics (3CPMFs), for the development of truly safe and comfortable ETs with enhanced thermal management.

## 2. Methods, Results and Discussion

**Figure 1:**
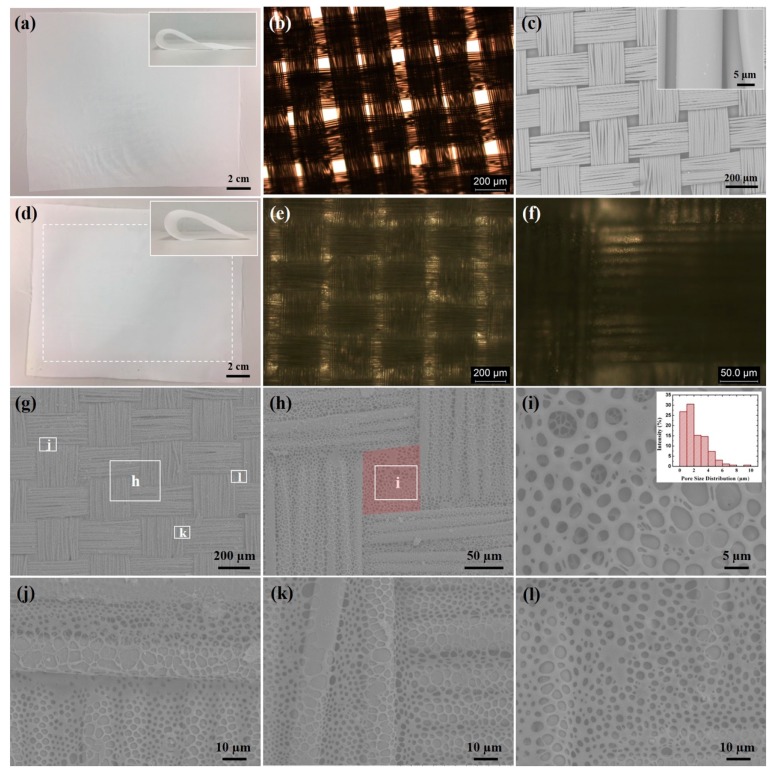
(**a**) Pristine PET fabric and its (**b**) OM and (**c**) SEM images; (**d**) Modified PET fabric after introduction of PS by the BF technique and its (**e**,**f**) OM images at different magnification and (**g**–**l**) at different magnification at different areas. The dash frame marked in (**d**) is the modified area of PET fabric; (**h**,**j**–**l**) are magnified views of correspondent areas marked with solid frames in (**g**), while (**i**) is the magnified view of marked area in (**h**); The insets of (**a**,**d**) show the flexibility of PET before and after treatment. The inset of (**c**) shows the close view of unmodified fibers, while the inset of (**i**) shows the pore size distribution.

A kind of 3CPMF was first prepared via the BF technique using only commercially available polystyrene (PS) and polyester (PET) fabric. They are one of the most widely used polymers and textiles. Figure 1a and its inset show a piece of 20 cm × 16 cm PET fabric that was used. It possesses a typical woven texture composed of PET fibers, as demonstrated in Figure 1b,c. In addition, periodic voids with lengths ranging from tens to hundreds of micrometers were found to exist among the latitude and longitude of fiber bundles. A close view in the inset of Figure 1c indicates that PET fibers have a smooth surface and their diameters are approximately 15 μm. To prepare 3CPMFs, PS was first dissolved in chloroform and then introduced on a PET fabric in a high humidity environment according to the polymer weight density of 8 × 10^−4^ g·cm^−2^. Figure 1d shows a piece of PET fabric after treatment, where the modified area is framed with dash lines. The inset of Figure 1d indicates little influence on the flexibility of PET fabric after modification, and it was found that this kind of 3CPMF shows no obvious surface changes with respect to pristine PET fabrics when observed by naked eyes. In fact, however, the periodic voids were found to be well filled by the introduced PS when viewed under an optical microscope (Figure 1e). The optical microscopic (OM) images at higher magnification indicated that the fibers were decorated with elaborate microstructures (Figure 1f). A more detailed investigation was further conducted using a scanning electron microscope (SEM). At low magnification, clear woven textures, identical with pristine PET fabrics, were also observed by SEM (Figure 1g). A higher magnified image reveals that the elaborate microstructures contouring to the texture profile of fabrics were honeycomb-like porous microstructures (Figure 1h). The magnifications at different areas demonstrate that they were found to form along the profile of fibers in both longitudinal and latitudinal directions regardless of their surface complexity, including their intersections with large height differences (Figure 1j,k). The large voids were also well filled with a honeycomb porous coverage (red highlighted area in Figure 1h,i,l). In addition, its pore size is distributed from hundreds of nanometers to several micrometers, as shown in the inset of Figure 1i.

The formation of honeycomb porous microstructures is owing to the templating role of water microdroplets, while the nucleation and formation of water microdroplets are caused by the rapid evaporation of solvent in a high-humidity environment [9,10,11,12]. Actually, its formation mechanism implies complicated transfers of heat and mass at the interfaces among water, solvent, and polymer. The finally formed microstructural features are the results of three mutually interactive and restrictive processes of (I) evaporation of solvent, (II) formation of BFs involving the nucleation and growth of water droplets, and (III) self-assembly of polymers with BFs as templates involving molecule aggregation, precipitation, and gelation [13,14,15].

The physical properties of PET fabrics before and after modification by the BF technique are summarized in Table 1. Comparing to 110.60 μm of pristine PET fabric measured under 6 gf cm^−2^ pressure, the thickness of modified PET fabric was slightly increased to 120.60 μm, and its density was also slightly increased from 0.557 to 0.578 g·cm^−3^. These small increases are mainly attributed to the introduction of micrometer-sized polymers. Thanks to the existence of honeycomb porous microstructures, the moisture permeability, a key factor on the breathable comfort of fabrics for perspiration, was changed slightly. This property can be quantitively analyzed by using the target fabric to seal a 50-mL beaker filled with 25 g calcium chloride and monitoring the total weight of the beaker placed in the saturated humidity environment with time. The experimental results showed that the moisture permeability of PET fabrics after modification was slightly decreased to 2.23 mg cm^−2^·h^−1^ from 2.43 mg cm^−2^·h^−1^. The dynamic air permeability of fabric was also maintained, but due to the coverage of large voids of fabric with nanometer/micrometer-size honeycomb porous structures, it was inevitably increased from 0.21 to 25.84 KPa·s·m^−2^ after modification. Thermal conductivity is another key parameter of fabrics. It was decreased from 3.640 × 10^−2^ W·m^−1^·K^−1^ for pristine PET fabrics to 3.575 × 10^−2^ W·m^−1^·K^−1^ for modified PET fabrics. The lower thermal conductivity indicates the enhanced thermal insulation of fabrics, which can be ascribed to the very low thermal conductivity of used PS and the introduction of porous microstructures. The thermal conductivity of PS ranges between 0.033–0.105 W·m^−1^·K^−1^, which is much lower than 0.15–0.17 W·m^−1^·K^−1^ of PET [16].

**Figure 2:**
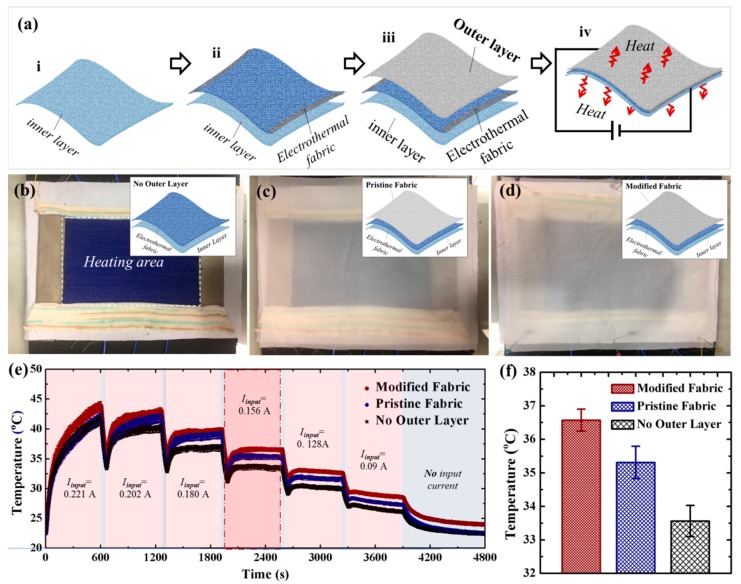
(**a**) Schematic process of assembling an ET device based on sandwiching an electrothermal fabric with an inner layer and an outer layer; ET devices with (**b**) no outer layer; (**c**) pristine PET fabric as outer layer; and (**d**) modified PET fabric by the BF technique as outer layer; (**e**) their electrothermal performance with time under different input power; and (**f**) electrothermal performance comparison under the same input current of 0.156 A.

Taking advantage of the enhanced thermal insulation and well-maintained moisture permeability, PS-modified PET 3CPMFs were used to assemble a kind of ET with a sandwiched structure, as illustrated in Figure 2. A piece of soft cotton fabric was firstly used as an inner layer and placed in the bottom (Figure 2a(i)). Then, another piece of electrothermal fabric knitted with conductive silver-plated yarns and wool yarn, which acts as a Joule heating unit, was placed in the middle (Figure 2a(ii)). Its electrical resistance, measured under constant tensile force, was approximately 64.8 Ω. Finally, pristine and modified PET samples were used as an outer layer to cover the heating unit (Figure 2a(iii)). The generation of heat can be achieved by applying a power supply and can be controlled by simply adjusting its input power (Figure 2a(iv)). The assembled ETs with no outer layer, pristine PET fabric as outer layer, and modified PET fabric by the BF technique as outer layer were shown in Figure 2b–d, respectively. Their structures were illustrated in the insets of Figure 2b–d, respectively. In addition, the effective heating regions were marked with a white dash frame in Figure 2b. To quantitatively analyze the electrothermal capacity of an ET, it was placed on an insulating foam in a control box with an ambient temperature of 21 ± 1 °C and relative humidity of 80 ± 5%. Six temperature sensors were attached in the center of the inner surface of the ET and placed in the middle of the ETs and the insulating form. They were used to monitor the temperature variation of ETs in real time. Figure 2e shows the temperature variation of different ETs with time under different power supplying states. To quickly find the thermal equilibrium, the input power first turned on for heating at a given input current for 10 min (highlighted in transparent red area), and then turned off for cooling for 1 min (highlighted in transparent blue area). The given input current gradually decreased from 0.221 to 0.090 A. It was found that the temperature curve of modified fabric-based ETs was always in a higher temperature than those of pristine fabric-based ETs and ETs without an outer layer, regardless of time and input current. The temperature curves of all ETs kept a rising trend within the given 10 min when the input current was over 0.180 A, while below 0.128 A they increased first and then showed a decreasing trend. The temperature curves were found to remain horizontal at 0.156 A, indicating the achievements of systematic thermal equilibrium. Figure 2f shows a comparison of the thermal equilibrium temperatures of different ETs at 0.156 A. It is obvious that modified fabric-based ETs can achieve a higher temperature than pristine fabric-based ETs under the same input power, which demonstrates the good thermal insulation performance of CPMFs during the Joule heating process. And the good thermal insulation of materials means their excellent thermal comfort provided for human beings.

**Figure 3:**
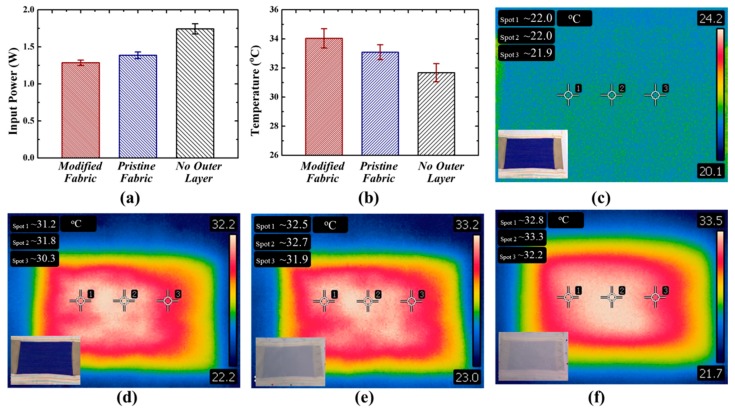
(**a**) The input power for different ETs to achieve the thermal equilibrium temperature of 34 °C; (**b**) The thermal equilibrium temperature of different ETs under the same input power of 1.29 W. Thermal images of ETs without outer layer (**c**) before and (**d**) after applying an input power of 1.29 W. Thermal images of (**e**) pristine fabric-based ET and (**f**) modified fabric-based ET after applying an input power of 1.29 W.

The power consumption of different ETs was further studied at the thermal equilibrium temperature of 34 °C, which is usually the skin temperature of a healthy man. As shown in Figure 3a, ETs without outer layer and pristine fabric-based ETs consumed 1.74 and 1.39 W to maintain the thermal equilibrium at 34 °C, while only 1.29 W was required for modified fabric-based ETs. It means that the PET fabrics used for ETs can save approximately 7.19% electric energy after introduction of three-dimensionally conformal porous microstructures using very few polymers by the BF technique. When fixing the input power at 1.29 W, we found that the thermal equilibrium temperatures for ETs with no outer layer and with pristine fabric can only be maintained at 33.1 and 31.7 °C, respectively (Figure 3b). Their thermal images were shown in Figure 3d–f. In contrast, a thermal image of ET, without applying an input power, was shown in Figure 3c. Correspondingly, the insets of Figure 3c–f are their regular photos. Thermal images visually demonstrated that the heating effects of all ETs after applying an input power were obvious. The measured temperatures at the center of ETs with no outer layer, pristine fabric, and modified fabrics were about 31.8, 32.7 and 33.3 °C, respectively. The temperature measured using an IR imaging system was close to the temperature monitored by temperature sensors for ETs without an outer layer, but for ETs with pristine fabric and modified fabric, the temperatures measured by the former were lower than those measured by the latter. One reason could be due to the various positions of ETs for measurement. Temperature sensors were embedded between the ETs and insulating foam for measuring the temperature of the inner surface, while the temperatures measured via IR imaging systems were at the outer surface of the ETs. Another reason can be ascribed to the thermal insulating properties of fabrics. The temperature difference caused by the pristine fabric was about 0.4 °C, while it was increased to 0.7 °C for modified fabric with three-dimensionally conformal porous microstructures of PS. These results indicate better thermal insulation of modified fabric than pristine fabric, which were well coincident with the results discussed above.

## 3. Concept of Demonstration

**Figure 4:**
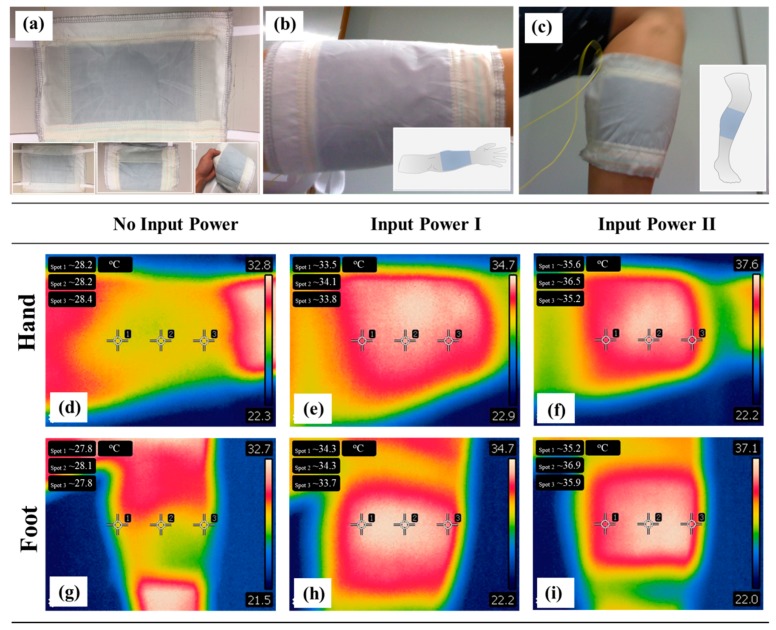
(**a**) Different digital photographs of modified fabric-based ETs after well sewing, and their wearable applications as (**b**) arm warmer and (**c**) leg warmer, respectively. Thermal images of arm warmer applied with an input power of (**d**) 0 W, (**e**) 1.5 W, and (**f**) 1.8 W, respectively. Thermal images of the leg warmer applied with an input power of (**d**) 0 W, (**e**) 1.5 w, and (**f**) 1.8 W, respectively.

Benefiting from the choice of bio-friendly polymers and fabrics only, 3CPMFs can be directly used for the fabrication of ET devices via stitching machine for safely wearable purposes. Figure 4a and its insets show different digital photographs of a kind of wearable ET warmer using PS-modified PET 3CPMFs as the outer layer. It can be applied as an arm warmer or leg warmer to be truly put on a human body (the human subject gave his informed consent for inclusion before he participated in the study) without concerns for safety and comfort, as shown in Figure 4b,c, respectively, and Figure 4d,g show their thermal images without input power, respectively. They can effectively warm up the covering area after applying an input voltage, and the temperature of the heating area can be adjusted as required by simply controlling the input power. For example, the temperature of the heating area can be increased to approximately 34 and 37 °C after applying an input power of 1.5 W (Figure 4e,h) and 1.8 W (Figure 4f,i), respectively. This active thermal management of ETs is of great use to combat different chilly weather.

## 4. Conclusions

In conclusion, we have experimentally demonstrated that the controllable surface modification of fabrics with three dimensionally conformal porous microstructures can effectively maintain the inherent properties of textile materials (i.e., fabric texture, flexibility, moisture permeability, and light weight) and introduce excellent performance of functional materials (i.e., good thermal insulation using polymers with low thermal conductivity). This kind of 3CPMFs was demonstrated to be good textile materials for the fabrication of flexible ET devices with both wearable comfort and enhanced thermal management. They exhibited better electrothermal and energy-saving performance than pristine fabric-based ET devices under the same conditions. Owing to the multitudinous choice of functional materials and textiles, various kinds of 3CPMFs with customized functionalities and properties can be designed and fabricated as required, which will lead to a brand-new kind of modified textiles. We believe that this work is of great significance to pave the way for the development of truly wearable devices with both desired functional performance and wearable comfort.

## Figures and Tables

**Table 1:** Comparison of the physical properties of PET fabrics before and after modification by the BF technique.

Sample	Thickness (μm)	Density (g·cm^−3^)	Moisture Permeability (mg·cm^−2^·h^−1^)	Thermal Conductivity (10^−2^ W·m^−1^·K^−1^)
**Pristine PET Fabric**	110.60 ± 0.49	0.557 ± 0.002	2.43 ± 0.14	3.640 ± 0.013
**Modified PET Fabric**	120.60 ± 1.20	0.578 ± 0.006	2.23 ± 0.14	3.575 ± 0.034
